# Effectiveness of the SARS-CoV-2 Vaccination in Preventing Severe Disease-Related Outcomes: A Population-Based Study in the Italian Province of Bolzano (South Tyrol)

**DOI:** 10.3389/ijph.2024.1606792

**Published:** 2024-03-14

**Authors:** Antonio Lorenzon, Lucia Palandri, Francesco Uguzzoni, Catalina Doina Cristofor, Filippo Lozza, Riccardo Poluzzi, Cristiana Rizzi, Pierpaolo Bertoli, Florian Zerzer, Elena Righi

**Affiliations:** ^1^ Department of Biomedical, Metabolic and Neural Sciences, University of Modena and Reggio Emilia, Modena, Italy; ^2^ Epidemiological Surveillance Unit, South Tyrolean Health Care Agency, Bolzano, Italy

**Keywords:** SARS-CoV-2 vaccination, vaccine effectiveness, COVID-19, hospitalisation, intensive care, severe disease, death, length of stay

## Abstract

**Objective:** To investigate the effectiveness of SARS-CoV2 vaccination in preventing ordinary or intensive care unit (ICU) admissions and deaths among cases registered during a variant transitional pandemic phase in the geographically and culturally unique territory of the Province of Bolzano (South Tyrol), an Italian region with low vaccination coverage.

**Methods:** We collected data from 93,643 patients registered as positive for SARS-CoV-2 by health authorities during the winter of 2021–22. The data were analyzed retrospectively using descriptive statistics and multiple logistic regression.

**Results:** 925 patients were hospitalized (0.99%), 89 (0.10%) were in intensive care, and 194 (0.21%) died. Vaccinated patients had a significantly lower risk of being hospitalized: adjusted Odds Ratio (aOR): 0.39; 95% CI: 0.33–0.46, ICU admission: aOR: 0.16; 95% CI: 0.09–0.29 and death: aOR: 0.41; 95% CI: 0.29–0.58. Similar risk reductions were also observed in booster-vaccinated patients, independent of sex, age, and predominant variant. Furthermore, the median length of stay (LoS) in the ICU was significantly longer for unvaccinated individuals compared to vaccinated subjects (9 vs. 6 days; *p* < 0.003).

**Conclusion:** Primary series vaccination and ongoing campaign booster doses were effective in preventing all severe disease-related outcomes and in reducing ICU Length of Stay, even during a transitional pandemic phase and in a unique territorial context.

## Introduction

The autonomous province of Bolzano, also known as South Tyrol, is the northernmost administrative region in Italy and is completely immersed in the Alps. Its mountainous landscape and proximity to the Austrian border make it a very complex territory, both geographically and socially. The population is mainly composed of Tyroleans, a traditionally German-speaking ethnic group, with two other consistent minorities featuring Ladins, an ancient population living in a few high alpine valleys, and Italians, who have recently settled in the region and especially in the cities. The territorial conformation is represented by small rural mountain valleys with a highly developed tourist economy, that converge into larger ones, where important trade routes connect Italy to Central Europe, and where the main settlements are located with relevant institutional centers and health facilities [1].

**FIGURE 1:**
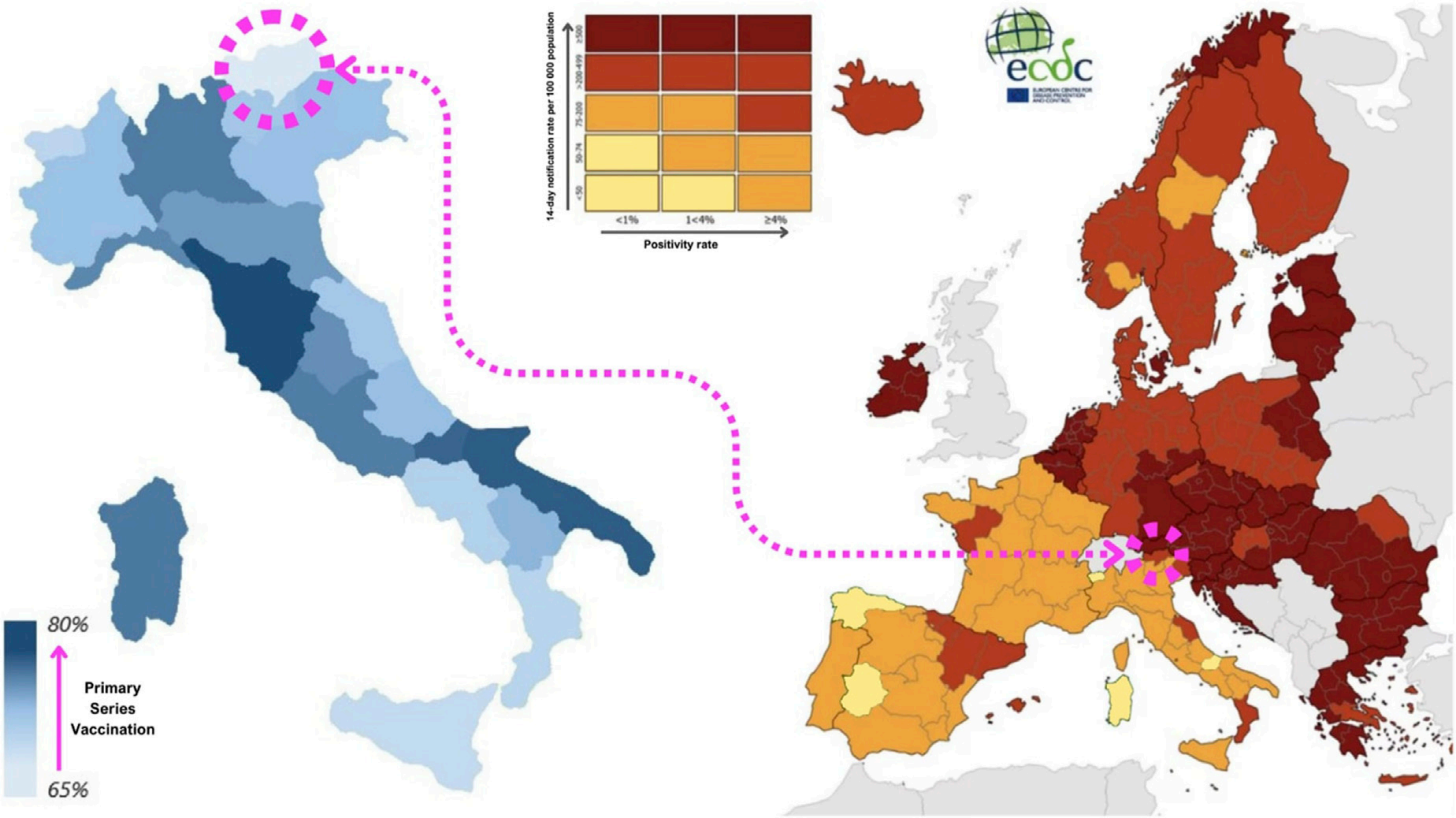
On the left side, map of completed primary series coverage between Italian regions on the second week of February 2022. (Italy, 2022) [2] On the right side, SARS-CoV-2 incidence rates between European territories on the first week of November (image taken and adapted from the European Centre for Disease Prevention and Control) [3]. (Europe, 2021).

The specificity of this territory has been maintained in the dynamics of the SARS-CoV-2 pandemic. As a matter of fact, South Tyrol showed indicator trends that were often unique and not in line with the rest of Italy or with the bordering countries beyond the Alps, such as Germany and Austria, with which it has high cultural and commercial exchange. For example, during the fall of 2021, the Delta SARS-CoV-2 variant affected Central Europe with a particularly violent wave, while Mediterranean countries were impacted with less intensity. However, South Tyrol had a much higher incidence than the Italian average, with nearly 200 cases per 100,000 people in early November 2021, compared with 62 cases per 100,000 in Italy (Figure 1). The even more infectious Omicron variant, on the other hand, hit the area during the following Christmas holidays with a timing and intensity similar to the national one [3, 4].

Although the incidence of SARS-CoV-2 reached its highest levels at that time, the winter of 2021–2022 was the first period without social restrictions in Italy, except for the use of personal protective equipment (PPE) and the requirement to be tested or vaccinated to take part in social activities [2]. Indeed, the SARS-CoV-2 vaccine played a key role in the resumption of activities during the pandemic, and even in this case the province of Bolzano showed unique characteristics, being simultaneously one of the regions with the most advanced booster vaccination campaign and the one with the lowest primary series coverage (Figure 1) [5].

At the beginning of February 2022, the Italian primary series vaccination coverage was 80%, compared with the South Tyrolean average of 66%, despite the great heterogeneity within the region, which again is proof of its complexity [5]. The booster dose vaccination campaign was initially reserved for the most fragile and high-risk individuals, and then gradually made available to everyone free of charge. From the beginning of November 2021 to 10 February 2022, while the primary series coverage of the South Tyrolean population increased by 8%, the booster dose coverage rose from 3% to 46%, although with strong variability according to age. In the 65+ age group, for example, coverage grew from 8% to 73%. This is much higher than the 52% booster dose coverage achieved by the 30–65 age group, which experienced steady growth, and even higher than that of the under-30s, who only started being administered boosters around Christmas time and reached 19% [6, 7].

Despite the consolidated evidence on the effectiveness of SARS-CoV-2 vaccines in preventing severe disease, literature on real-world data collected during specific pandemic periods, such as those characterized by the spread of the new variants or the onset of a booster campaign, is scarce. In addition, evidence is lacking on the actual effectiveness of vaccination campaigns in areas characterized by specific epidemiological, cultural, or geographical characteristics, making the province of Bolzano an ideal study setting. As suggested by the literature, analysis of data from the region should confirm a reduced risk of encountering a worse outcome, such as hospitalization, ICU admission, or death, in patients with higher vaccination status [8–10].

Therefore, our study started with some research questions: Was the risk of severe disease-related outcomes significantly reduced by vaccination, even in such a unique context? Could positive effects of the booster dose on outcomes already be observed, while the booster administration campaign was still ongoing?

The aim of the study is to describe the effectiveness of vaccination against COVID-19 (primary series or booster dose) in preventing severe disease-related outcomes in a specific territory and during a transitional phase of the pandemic.

## Methods

A population-based epidemiologic study was conducted retrospectively using health data extracted from the surveillance system database covering the entire territory of South Tyrol and referring to the winter season 2021–22.

The information used for the study came from the Bolzano Epidemiological Surveillance Unit of the Local Health Authority (Südtiroler Sanitätsbetrieb—Azienda Sanitaria dell’ Alto Adige) and was collected to track SARS-CoV-2 cases in the territory. One of the tasks of the unit was to collect pandemic data from the entire area and process both aggregated and individual data streams, addressed to the press and the Ministry of Health, thus constituting the official data source of the local health authority.

For our study, we extracted data on new diagnoses of the resident population that occurred between 1 November 2021 and 12 February 2022 and were reported to the Ministry of Health, as part of the COVID-19 pandemic health surveillance. Information on hospitalization and death collected up to 21 days (by 4 March) after diagnosis was added to these cases.

The study protocol was approved by the Ethics Committee of the Hospital of Bolzano on 16 March 2022 (Prot. 0259655-BZ).

### Inclusion and Exclusion Criteria

The inclusion criterion for SARS-CoV-2 cases was that they had been reported as a new case of SARS-CoV-2 to the Ministry of Health by the Health Authority of the Province of Bolzano between 1 November 2021 and 12 February 2022. This category includes all patients who took a diagnostic swab with a positive result at an authorized center without a recorded positive result in the previous 90 days [11].

Only cases residing in South Tyrol were included in the study because vaccination information for the non-resident population might be missing from the database.

For each subject included in the study, we collected main demographic information (age, sex, district of residence); vaccination status (vaccine type, dose, date of vaccination), and characteristics of the infection course, including the date of the first positive swab, hospitalization (date of admission and discharge, general or intensive care ward) and death (yes/no and date) that occurred in the 21 days following the date of the first positive swab (until 4 March 2022) or after a previous hospitalization during the infection.

Only deaths caused by SARS-CoV-2 were recorded, while hospitalizations were excluded if they occurred in ordinary wards under either of these two conditions:

• Patients admitted in a ward not designed for COVID-19 hospitalization and no symptomatic COVID-19 diagnosis code reported: ICD10 *043.1*, *043.2*, and all *480* subcategories [12].
• Patients in wards designated for COVID-19, for which some diagnosis codes were reported, but none indicating the symptomatic infection.

Information on the prevalent variant, obtained from the weekly reports of the Public Health Laboratory and the Health Authority, was collected to investigate its role as a covariate. All vaccinations whose validity was not recognized by Italian Law at the time of the study were excluded [13].

### Measurements

Vaccination against SARS-CoV-2 was considered the primary exposure. Vaccination status was summarized in a single categorical variable (*vaccination status*) with three immunization groups (no vaccination/primary series/booster dose). In accordance with national guidelines and scientific evidence, a previous infection that occurred at least 90 days prior to the current one was considered a new positive and was ranked within the immunization groups as equivalent to a single dose of vaccination. Therefore, the presence of a previous infection along with one or two doses of vaccine constitutes the completion of the primary series or booster vaccination [14–16].

More specifically

• “Unvaccinated” included all subjects who did not complete or even start the first vaccination cycle, regardless of the number of previous infections.
• The primary series included all patients who, at the time of diagnosis, had correctly completed the primary series of a vaccine recognized by the Italian Ministry of Health, but not a booster or additional dose. This included:
  Two doses of Comirnaty (Pfizer/BioNTech) or Moderna, given between 15 and 42 days apart; or two doses of Vaxzevria/AstraZeneca, given between 70 and 84 days apart. All three vaccines had to be given at least 15 days before diagnosis [17].
  Other vaccines approved by the EU [13].
  One dose of Johnson & Johnson (manufactured by Janssen) at least 15 days before diagnosis [18].
  One dose of vaccine and a documented infection between 14 and 180 days after vaccination; or a prior infection and a vaccine dose taken from 3 to 12 months later [19, 20].
• Booster patients are defined as those with an additional or booster dose of any of the licensed vaccines, or an infection that occurred at least 5 months after the primary series and 4 months after the last infection [19, 21].

Furthermore, the variable named *Days since the last dose,* available only for vaccinated patients, was created by reporting the number of days elapsed since the last dose (booster or primary series) on the day of diagnosis.

Variables related to sex, age (at diagnosis), and predominant viral variant during the week of the positive swab were considered covariates, as they are known to be associated with COVID-19-related outcomes [10, 22].

Some of these variables were grouped into categories:

• Age was categorized as 0–29, 30–65, and over 65 years according to the United States Department of Health and Human Services guidelines, which indicate a 97-fold higher risk of death for category 3 compared to category 1 [23, 24].
• Weekly data on viral variant prevalence, extracted from viral RNA samples obtained from nasopharyngeal swabs and extracted by PCR methodology by the Public Health Laboratory of Microbiology and Virology, were grouped into three categories based on the percentages detected in each week’s total sampling. *Delta week* if this variant was present in more than 90% of the samples, *Omicron* week if this variant was present in more than 90% of the samples, and *Transition week* in all other cases. This categorization resulted in three different time ranges in which the other events took place. The first one corresponded to the *Delta period* and ran from the beginning of the study until 17 December. This was followed by the *Transition Period*, which lasted 4 weeks, until 14 January, and gave way to the *Omicron period*, which lasted until the end of the study [25].

The severity of the infection course, specifically hospitalization (general or intensive care) and death were considered as primary outcomes:

• Hospitalization: refers to the stay (yes/no) of a positive swab subject in one of the hospitals equipped to treat COVID-19 patients. These were located in Merano, Bolzano, Bressanone, Brunico, Silandro, Vipiteno, and San Candido. The wards equipped to receive these patients, in addition to the dedicated ward set up in the hospital of Bolzano, were intensive/sub-intensive care and ordinary wards, such as pediatrics (and neonatology), geriatrics, internal medicine, and emergency (casualty ward).
• Admission to an intensive care unit (ICU): refers to the stay (yes/no) of a positive case in one of the four intensive and sub-intensive care units in the province of Bolzano, located in the hospitals of Bressanone, Brunico, Merano, and Bolzano.
• Death: refers to the death (yes/no) of positive swab patients reported as symptomatic for COVID-19 infection.

Additionally, the length of stay (LoS), both overall and in the ICU, was determined by calculating the time (in days) elapsed between admission and discharge or death. In the same way, the days elapsed between diagnosis and hospital admission were estimated.

Full information on the variables and potential risks of bias can be found in the Supplementary Material.

### Statistical Analysis

Categorical variables were summarized using absolute and relative frequencies. The median and interquartile range (IQR) were used to summarize continuous variables due to their non-normal distribution. Pearson’s chi-squared test was used to compare categorical variables. The Mann–Whitney U-test was used to analyze numerical variables according to their distribution. Multiple logistic regression models were constructed to examine the association of vaccination status with hospitalization, ICU admission, or death due to COVID-19. The summary variable *Vaccination status* was used as the primary exposure variable in the model. To control for potential confounders and evaluate their role, covariates were also included in the model. Associations are reported as adjusted Odds Ratios (aOR) and 95% confidence intervals (95% CI).

A second logistic regression model including days since last dose was run only for vaccinated patients was used to assess the time-effectiveness of vaccines.

Finally, vaccine effectiveness was calculated using the formula: $VE=\left(1-odds\;ratio\right)*100$

SPSS Statistics, version 27, and Excel, version 16.0, were used to perform the analyses [26].

This study was reported according to the Strengthening the Reporting of Observational Studies in Epidemiology (STROBE) guidelines [27].

## Results

We collected data on 93,642 patients who tested positive for SARS-CoV-2; the main characteristics of the entire sample, overall and stratified by vaccination status, are described in Table 1. In total, slightly more cases occurred in women (52%), in the 30–65 age group (48%) and during the Omicron period (67%). Slightly more than half of the cases were unvaccinated (53%), one-third were vaccinated with a primary series (33%), and one-sixth (15%) with a booster dose.

**TABLE 1:** Main characteristics and outcomes of the total sample according to vaccination status (South Tyrol, Italy, 2021–2022).

Sample	Characteristics	Total	Vaccination status			*p*-value
			Unvaccinated	Primary series	Booster dose	
Overall		93,642	49,176 (52.51%)	30,581 (32.66%)	13,885 (14.83%)	
Sex	Male subjects	44,996 (48.05%)	24,119 (49.05%)	14,622 (47.81%)	6,255 (45.05%)	
	Female subjects	48,646 (51.95%)	25,057 (50.95%)	15,959 (52.19%)	7,630 (54.95%)	<0.001
Age	Under 29	41,899 (44.74%)	27,821 (56.57%)	11,857 (38.77%)	2,221 (16%)	
	Adults 30–65	44,989 (48.04%)	19,416 (39.48%)	16,377 (53.55%)	9,196 (66.23%)	
	Over 65	6,754 (7.21%)	1,939 (3.94%)	2,347 (7.67%)	2,468 (17.77%)	<0.001
	Years	33 (16–50)	23 (9–45)	36 (22–50)	47 (36–60)	<0.001
Variant	Delta	16,905 (18.05%)	10,799 (21.96%)	5,625 (18.39%)	481 (3.46%)	
	Transition Period	13,831 (14.77%)	6,925 (14.08%)	5,468 (17.88%)	1,438 (10.36%)	
	Omicron	62,906 (67.18%)	31,452 (63.96%)	19,488 (63.73%)	11,966 (86.18%)	<0.001
Hospitalization	No	92,717 (99.01%)	48,670 (98.97%)	30,317 (99.14%)	13,730 (98.89%)	
	Yes	925 (0.99%)	506 (1.03%)	264 (0.86%)	155 (1.11%)	0.018
ICU^a^ admission	No	93,553 (99.90%)	49,111 (99.87%)	30,567 (99.96%)	13,875 (99.93%)	
	Yes	89 (0.10%)	65 (0.13%)	14 (0.04%)	10 (0.07%)	<0.001
Death	No	93,448 (99.79%)	49,077 (99.80%)	30,515 (99.78%)	13,856 (99.79%)	
	Yes	194 (0.21%)	99 (0.20%)	66 (0.22%)	29 (0.21%)	0.907
LoS	Days in the ICU^a^	7 (4–12)	9 (5–19)	4 (2–7)	7 (5–14)	0.022
	Length of hospitalization (days)	7 (4–13)	7 (4–14)	8 (4–13)	7 (4–12)	0.793
Other time ranges	Days between Diagnosis and Hospitalization	1 (0–5)	2 (0–7)	0 (0–3)	0 (0–1)	<0.001
	Days since last vaccination Dose	112 (48–166)	—	147 (103–181)	42 (18–59)	<0.001

Numbers are presented as n (%) or median (IQR).

^a^ Intensive Care Unit.

Within the positive swap population of the study, differences in age and sex prevalence were observed between vaccination groups, as well as in the frequency of vaccination status across variant periods.

A total of 925 subjects (0.99% of the sample) were hospitalized, 89 (0.10%) were admitted to an ICU ward and 194 (0.21%) died. Differences in severity of the disease between unvaccinated, vaccinated, and booster subjects were significant mainly for ICU admission, which showed a frequency equal to 0.13%, 0.04%, and 0.07% for subjects unvaccinated, with the primary series completed and with a booster dose, respectively (*p*-value < 0.001).

Considering the median length of stay in the intensive care unit of the overall population, unvaccinated patients stayed in the ICU 5 days longer than those vaccinated with the primary series, and 2 days longer than those with the booster, while the overall length of hospitalization was similar in the three categories (1 day difference). The distribution by age and variant is shown in Supplementary Table S1.

The median number of days elapsed between diagnosis and hospitalization was 2 for the unvaccinated and 0 for both vaccinated categories (*p*-value < 0.001). The primary Series and Booster dose groups differed in the number of days elapsed between the last dose administered and the day of diagnosis: 147 (103–181) for the primary series, and 42 (18–59) for the booster (*p*-value < 0.001).

Table 2 describes the COVID-19-related outcomes in relation to vaccination status, sex, age, and variant prevalence.

**TABLE 2:** Severity of infection in the entire sample among covariates and time since the last vaccine dose (South Tyrol, Italy, 2021–2022).

Exposition	Outcome	Hospitalization			ICU^a^ admission			Death		
		No	Yes	*p-*value	No	Yes	*p-value*	No	Yes	*p-*value
Sex	M	44,471 (98.83%)	525 (1.17%)		44,943 (99.88%)	53 (0.12%)		44,887 (99.76%)	109 (0.24%)	
	F	48,246 (99.18%)	400 (0.82%)	<0.001	48,610 (99.93%)	36 (0.07%)	0.03	48,561 (99.83%)	85 (0.18%)	0.023
Age	Median	33 (16–49)	75 (60–84)	<0.001	33 (16–50)	66 (56–78)	<0.001	33 (16–50)	85 (78–90)	<0.001
Age	under 30	41,848 (99.88%)	51 (0.12%)		41,897 (99.96%)	2 (0.01%)		41,899 (100%)	0 (0%)	
	30–65	44,741 (99.45%)	248 (0.55%)		44,950 (99.91%)	39 (0.09%)		44,980 (99.98%)	9 (0.02%)	
	over 65	6,128 (90.73%)	626 (9.27%)	<0.001	6,706 (99.29%)	48 (0.71%)	<0.001	6,569 (97.26%)	185 (2.74%)	<0.001
Variant	Delta	16,487 (97.53%)	418 (2.47%)		16,846 (99.65%)	59 (0.35%)		16,801 (99.39%)	104 (0.62%)	
	Transition Period	13,684 (98.94%)	147 (1.06%)		13,814 (99.88%)	17 (0.12%)		13,805 (99.81%)	26 (0.19%)	
	Omicron	62,546 (99.43%)	360 (0.57%)	<0.001	62,893 (99.98%)	13 (0.02%)	<0.001	62,842 (99.90%)	64 (0.10%)	<0.001
Days from last vaccination dose^b^	Median	111 (47–166)	140 (66–223)	<0.001	112 (48–166)	124 (70–257)	<0.001	111 (47–166)	210 (95–261)	<0.001

^a^ Numbers are presented as n (%) or median (IQR) as appropriate.Intensive Care Unit.

^b^ Relating only to the 44,466 patients who received the COVID-19 vaccination.

Disease severity showed significant differences in relation to covariates. Outcomes were significantly higher in male patients than in female patients, occurred more frequently during the Delta period, increased with age, and, if vaccinated, also increased with the number of days since the last dose. Outcomes also varied by age and variant stratified together, as shown in Supplementary Table S2.

**FIGURE 2:**
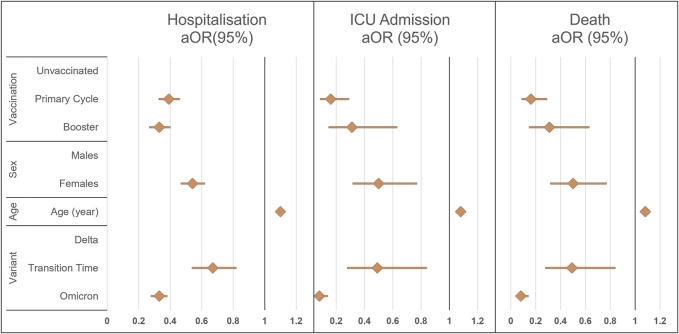
Results of the multiple logistic regression model for the three different outcomes of disease severity, overall hospitalization, Intensive Care Unit admission, or death. ICU, intensive care unit; aOR (95%), adjusted odds ratio with 95% confidence intervals (South Tyrol, Italy. 2021–22).

Figure 2 shows the results (aOR and 95% CI) of the multiple logistic models analyzing the 3 main COVID-19 outcomes evaluated in the study in relation to vaccination status, age, sex, and prevalent variant.

Both primary series and booster-vaccinated subjects had a reduced probability of hospitalization, ICU admission, or death compared with unvaccinated patients. Reduced odds for all outcomes were also observed in female patients, in those of younger age, and during the Transitional and Omicron periods. Adjusted Odds Ratios are shown in Supplementary Table S3.

According to the results of multiple logistic regression, the vaccine effectiveness (VE) for preventing hospitalization was found to be 61% (67%–54%) for the primary series and 67% (60%–73%) for the booster dose; for preventing ICU admission 84% (71%–91%) for the primary series and 69% (37%–85%) for the booster dose, and for preventing death, 59% (42%–71%) for the primary series and 79% (67%–86%) for the booster dose.

The results of the second logistic model showed that the probability of the three primary outcomes also increased with time since vaccination; by 0.3% for hospitalization, 0.2% for ICU admission, and 0.5% for death for each additional day since the last dose, as shown in Supplementary Table S4.

## Discussion

### Key Results

This study analyzed real-world data from the winter of 2021–22 in the Italian province of Bolzano, also known as South Tyrol, with the main aim of evaluating the effectiveness of vaccination against severe COVID-19 disease during a transitional pandemic phase.

Consistent with worldwide data, our findings showed significant differences in the probability of hospitalization, admission to intensive care, or death according to sex, age, and circulating variant [28–30]. The odds of experiencing these outcomes increased by approximately 50% in male patients, decreased from Delta variant to Omicron (by approximately 70% for death and hospitalization, more than 90% for ICU admissions), and increased by approximately 10% for each additional year of age (18% for death).

Despite the significantly lower vaccination coverage compared to the rest of Italy, the protective effect of the vaccines was significant [5]. In this study, among subjects who completed the primary vaccination cycle, the probability of being hospitalized or dying was nearly one-third when compared to that of unvaccinated subjects, and more than one-fifth when considering ICU admissions. Even the booster dose, although the campaign was ongoing and started by targeting the most vulnerable individuals, was shown to reduce the probability of COVID-19-related outcomes compared to the unvaccinated.

Unlike other similar studies, we had the opportunity to evaluate the length of hospital stay both in regular wards and the ICU. Overall, the average LoS in regular wards showed only small differences. However, LoS in the ICU was significantly longer for unvaccinated patients (median LoS: 9 days) in comparison to primary series (4 days) and booster (7 days) vaccinated patients. Notably, the probability of experiencing the primary outcomes increased by shifting temporally away from vaccination.

Furthermore, the median number of days elapsed between the last dose of vaccine and the detection of SARS-CoV-2 was interestingly long: 147 days after completion of the primary series and 42 days for those who also received a booster dose. The coincidence of vaccination with a period of high viral circulation suggests that some factors may have reduced the risk of contagion in the weeks immediately after dose administration, such as the vaccination itself, as suggested by previous studies [31, 32].

### Interpretation and External Validity

In conclusion, the findings of our study support vaccination against SARS-CoV-2, in line with other population studies and systematic reviews [33–35].

The results of this study confirmed the ability of vaccines to prevent severe COVID-19 disease, even in such a unique context. Vaccine effectiveness ranged, depending on the outcome considered, from 59% to 84% for the primary series and 67%–79% for the booster dose. These are slightly lower than those reported in other national and international studies. Data coming from the Apulia region, for example, showed vaccine effectiveness of more than 90% in preventing hospitalization and death during the Alpha to Delta period, while a study carried out in the UK during the Delta period only estimated it at 71%–84% [36, 37]. Probably, the lower protection observed in our results—referring to the Delta to Omicron period—is due precisely to the variants considered. In fact, a study conducted in the UK during the Omicron period found an even lower effectiveness against symptomatic disease, ranging from 75.1% 2–4 weeks after vaccination to 14.9% at 2 years [38]. In this context, the time elapsed since vaccination also seems to have determined a progressive loss of effectiveness, as widely described in the literature and confirmed by our data [36, 38].

However, the potentially diminished protective effect of the vaccine against new variants was also suggested by a reduction, shifting from the Delta to the Omicron period, in the difference in outcome frequency between vaccinated and unvaccinated. Together with the loss of vaccine protection over time, this underlines the necessity to maintain immunity with updated booster doses of new variants [10]. The limited diagnoses in the first weeks after booster inoculation observed in our study, also support this administration policy in order to reduce the virus circulation [31, 32].

Furthermore, our results evidenced a shorter LoS for vaccinated patients compared to unvaccinated patients, suggesting a more rapid attenuation of severe symptoms, and providing insight for future research on an aspect that is still understudied and the subject of scientific debate [39, 40]. Overall, this study contributes valuable information to the existing literature.

### Limitations and Strengths

The results of the present study must be read in light of their limitations. First, a possible underdiagnosis could have occurred, especially in the vaccinated group, since during the study period unvaccinated subjects had to routinely test negative to obtain a green pass to work or participate in most social activities. This theory is supported by the difference, between vaccinated and unvaccinated, in the days between diagnosis and hospitalization: those who were vaccinated were on average tested positive on the same day of hospital admission, while those who were unvaccinated tested positive 2 days before. Consequently, this underdiagnosis could also lead to an overestimation of the hospitalization and mortality rates in the vaccinated groups and therefore to an underestimation of vaccine effectiveness. In contrast, the number of COVID-19-positive hospitalizations and deaths is not affected by this limitation because all hospitalized and deceased subjects were tested [2].

Another limitation of the study is the lack of information on the symptomatology and comorbidities of the patients, which makes it difficult to ascertain the quantitative contribution of COVID-19 to the outcome itself. Moreover, the study has the inherent limitation of not being able to capture the overall development of the pandemic as it covers a brief period compared with the entire course of the pandemic and the vaccination campaign. For the same reason, since the most vulnerable individuals, and therefore those most at risk of negative outcomes, were enrolled first during the vaccination campaigns, the booster group is not perfectly comparable with those who were vaccinated with the primary series, whose vaccination campaign was more advanced, nor with the pediatric unvaccinated, who had just had their first access to vaccination. Finally, the days elapsed since the last dose were only available for the vaccinated [6, 7].

However, this study also has many strengths. First, our results rely on a large sample size which is valuable considering all the difficulties in data collection during the first period of the pandemic. Furthermore, the Italian healthcare system is predominantly public and there should be no lack of admissions. Finally, the results were consistent with each other and with the existing literature, a sign of the reliability of the data collected and the correctness of the methods used.

### Conclusion

The implications of the study are multifaceted. First, it confirms the effectiveness of SARS-CoV-2 vaccines in a large sample, and with a high proportion of unvaccinated patients, especially in preventing severe outcomes in the elderly. It also suggests an effect on reducing the duration of hospitalization and preventing contagion. The study also confirms the decreasing risk of severe disease passing from the Delta to the Omicron variant, although the absolute frequencies of outcomes were similar due to the larger number of cases.

By proving the effectiveness of both the primary series and the booster dose in a region characterized by low vaccination coverage and high hesitancy, our research suggests an estimate of the health benefits, and cost savings due to the reduction in the number and duration of hospitalizations that can be obtained and achieved through the vaccine and booster campaigns. This supports the policy of administering booster doses, whose effectiveness is also proven compared to primary series vaccination, whose protection tends to decline over time.

Future studies should focus on larger samples of hospitalized patients to further investigate the length of hospital stay (LoS), as this study provides promising evidence of a shorter duration for vaccinated individuals. In addition, data on comorbidities and symptomatology should be recorded as these would allow for the building of more robust and informative models. Finally, further research could focus on a deeper analysis of this unique territory, also using different methodological approaches, to better understand both the epidemiology of the disease and vaccination adherence.

Overall, this study contributes to the growing body of evidence supporting the importance of vaccination in mitigating the impact of COVID-19, providing solid and consistent results on the one hand, and insights for future research on the other.
