# Supplementary material for: Effectiveness of the SARS-CoV-2 Vaccination in Preventing Severe Disease-Related Outcomes: A Population-Based Study in the Italian Province of Bolzano (South Tyrol)
Source: Int J Public Health. 2024 Mar 14;69:1606792. doi: 10.3389/ijph.2024.1606792 (PMC10976940; doi:10.3389/ijph.2024.1606792)
Supplement: Supplementary file 1 [file DataSheet1.DOCX]

**Supplementary Material**

**Additional information on variables**

The data on new positives of the day came from all facilities that regularly practised nasopharyngeal swabs. As of 1^st^ November 2022, the date coinciding with the start of the study period, both naso-pharyngeal swabs and PCRs had diagnostic power. One day later, nasal swabs also acquired it, which before then had to be confirmed by a positive PCR performed as soon as possible.

Once the facilities (hospital departments, pharmacies, and general practitioners) registered a positive swab, it was uploaded on a specific portal of the health authority. At this point, the IT department carried out a personal reconciliation by using the fiscal code, which allowed the positive patients to be linked to the registry information contained in the province's administration databases. Information on both vaccination and residence/ domicile was retrieved, because based on this there would have been indication on time and location of quarantine.

Reports of deaths, however, came to the unit via manual reports to a dedicated mailbox. These were linked to the health information and checked one by one to add comorbidities and establish whether covid contributed to the death, before reporting it the press and adding the death to the dataset with the positive cases.

The data on hospitalisations, on the other hand, were obtained from the IT portal of the South Tyrol health authority, by using a search query created specifically for the unit. Information on admissions was therefore also added to the cumulative database of positive patients, where exclusion criteria were applied. This data was used by the unit to produce reports and indicators of outbreaks, and after a set period the oldest fields were gradually deleted.

Data on the population's vaccination adherence rate, originated from the prevention department's IT section, and reports on variants recorded by molecular tests from the microbiological laboratory, by other side, reached the unit weekly to monitor the spread characteristics of the virus and the protection of the population.

**Risk of bias**

Firstly, patients vaccinated outside the region who did not send the inoculation proof to the South Tyrolean prevention department, may be incorrectly categorized in a "lower" vaccination category. For this reason, all non-residents have not been included. However, this bias is likely to be unimportant for resident patients for several reasons. First, South Tyrol was quite advanced in its vaccination campaign related to other regions, so it was uncommon to vaccinate elsewhere. Secondly, at that time, travelling was still not recommended, further reducing the probability of people seeking vaccinations outside the region.

Another sampling bias could be produced by healthcare workers, who may not have reported the admission or the death of Covid-19 patients to the unit, especially during periods of high workload. This information may be completely missing, as well as that of residents who received a diagnosis and/or incurred an outcome outside the province of Bolzano. However, the large sample of patients should make this missing information irrelevant. Furthermore, this could be partially compensated by included individuals for whom Covid-19 contributed truly little to encountering the outcome.

Additionally, despite in Italy the healthcare system is predominantly public and there should be no shortage of admissions, some hospitalized patients may be missing due to the lack of a diagnostic code, when temporary placed in any non-Covid ward. However, this could be counterbalanced by those who, staying in a Covid-19 equipped department with no diagnostic codes at all, might be incorrectly included in the sample (e.g. paediatric or geriatric patients with significant comorbidities for whom COVID-19 had a negligible impact).

On the other side, information on variants may not be representative or slightly delayed as the analysis of PCR samples from patients may not have kept pace with the arrival of Omicron in the region, when antigen testing was much more common. In any case, PCR tests were mainly reserved for international travellers and hospital or socio-assistance settings, thereby covering both patients who underwent outcomes and who are likely to introduce a new variant.

Lastly, the number of positive cases may be incomplete as not all PCR tests conducted at pharmacies or private structures, if samples were sent to private laboratories abroad, were reported. Fortunately, few structures are estimates to have sent samples oversea. Moreover, vaccinated patients did not need to undergo regular testing for work or school, unlike unvaccinated individuals, who are expected to have a higher number of asymptomatic cases. The lack of information on symptoms makes it difficult to estimate the entity of this bias.

**Supplementary Tables**

Supplementary table 1: median of numeric variables and differences among age and variant categories. Numbers are presented as median (IQR). ^a^Intensive Care Unit

|  | *Median (IQR)* | Days between Diagnosis and Hospitalisation | Hospitalisation  length (days) | Days in ICU^a^ | Days from last Dose |
| --- | --- | --- | --- | --- | --- |
| Overall |  | 1 (0 - 5) | 7 (4 - 13) | 7 (4 - 14) | 112 (48 - 166) |
|  | Under 30 | 0 (0 - 2) | 3 (2 - 7) | 16.5 (1 - 32) | 119 (50 - 157) |
| Age Level | 30-65 | 2.5 (0 - 7) | 7 (4 - 13) | 10 (6 - 22) | 108 (46 - 170) |
|  | Over 65 | 0 (0 - 4) | 8 (5 - 14) | 6 (3 - 9.5) | 96.5 (51 - 181) |
|  | *p-value* | **<0.001** | **<0.001** | **0.009** | **<0.001** |
|  | Delta 90% | 1 (0 - 5) | 8 (4.5 - 15) | 8 (4 - 14) | 151 (115 - 182) |
| Variant | Intermediate | 1 (0 - 6) | 8 (4 - 15) | 7 (5 - 19) | 139 (64 - 176) |
|  | Omicron >90% | 0 (0 - 5) | 7 (4 - 12) | 6 (4 - 8) | 90 (43 - 155) |
|  | *p-value* | 0.101 | **0.002** | 0.62 | **<0.001** |

Supplementary table 2. Absolute risk of outcomes for each vaccination status, subdivided for the three variant periods. Numbers are presented as positive cases. ^a^Intensive Care Unit

|  | **Delta** |  |  |  | **Transition Time** | |  |  | **Omicron** |  |  |  |
| --- | --- | --- | --- | --- | --- | --- | --- | --- | --- | --- | --- | --- |
| *Hospitalisation* | Not hospitalised | Hospitalised | Absolute Risk | p-value | Not hospitalised | Hospitalised | Absolute Risk | p-value | Not hospitalised | Hospitalised | Absolute Risk | p-value |
| Not immunised | 10541 | 258 | 2.4% |  | 6831 | 94 | 1.4% |  | 31298 | 154 | 0.5% |  |
| Primary Cycle | 5492 | 133 | 2.4% | <0.001 | 5436 | 32 | 0.6% | <0.001 | 19389 | 99 | 0.5% | <0.001 |
| Booster | 454 | 27 | 5.6% |  | 1417 | 21 | 1.5% |  | 11859 | 107 | 0.9% |  |
| *ICU*^a^ *admission* | Not admitted | ICU admitted |  |  | Not admitted | ICU admitted |  |  | Not admitted | ICU admitted |  |  |
| Not immunised | 10754 | 45 | 0.4% |  | 6910 | 15 | 0.2% |  | 31447 | 5 | 0.0% |  |
| Primary Cycle | 5616 | 9 | 0.2% | 0.001 | 5466 | 2 | 0.0% | 0.006 | 19485 | 3 | 0.0% | 0.203 |
| Booster | 476 | 5 | 1.0% |  | 1438 | / |  |  | 11961 | 5 | 0.0% |  |
| *Death* | Healed | Dead |  |  | Healed | Dead |  |  | Healed | Dead |  |  |
| Not immunised | 10743 | 56 | 0.5% |  | 6909 | 16 | 0.2% |  | 31425 | 27 | 0.1% |  |
| Primary Cycle | 5583 | 42 | 0.7% | 0.041 | 5462 | 6 | 0.1% | 0.213 | 19470 | 18 | 0.1% | 0.092 |
| Booster | 475 | 6 | 1.2% |  | 1434 | 4 | 0.3% |  | 11947 | 19 | 0.2% |  |

Supplementary table 3. Multiple Logistic Regression Model for the three different outcomes considered. *Nagelkerke R^2. ^a^Intensive Care Unit

| Outcome: |  | Hospitalisation |  |  | ICU^a^ Admission |  |  | Death |  |
| --- | --- | --- | --- | --- | --- | --- | --- | --- | --- |
|  |  | (R^*^ =0.321) |  |  | (R^*^ =0.249) |  |  | (R^*^ =0.464) |  |
| Predictors | aOR | 95% C.I. | *p-value* | aOR | 95% C.I. | *p-value* | aOR | 95% C.I. | *p-value* |
| Primary series completed vs Not | 0.39 | (0.33 - 0.46) | 0.000 | 0.16 | (0.09 - 0.29) | 0.000 | 0.41 | (0.29 - 0.58) | 0.000 |
| Booster vs Not vaccinated | 0.33 | (0.27 - 0.40) | 0.000 | 0.31 | (0.15 - 0.63) | 0.001 | 0.21 | (0.14 - 0.33) | 0.000 |
| Gender: Females (vs Males) | 0.54 | (0.47 - 0.62) | 0.000 | 0.50 | (0.32 - 0.77) | 0.002 | 0.41 | (0.30 - 0.56) | 0.000 |
| Age (each more year) | 1.10 | (1.10 - 1.11) | 0.000 | 1.08 | (1.07 - 1.09) | 0.000 | 1.18 | (1.16 - 1.19) | 0.000 |
| Variant: Intermediate (vs Delta) | 0.67 | (0.54 - 0.82) | 0.000 | 0.49 | (0.28 - 0.84) | 0.011 | 0.62 | (0.39 - 0.99) | 0.048 |
| Variant: Omicron (vs Delta) | 0.33 | (0.28 - 0.38) | 0.000 | 0.08 | (0.04 - 0.14) | 0.000 | 0.29 | (0.20 - 0.41) | 0.000 |

Supplementary table 4. Multiple Logistic Regression Model for the three different outcomes, considering the time elapsed from last dose (in days). *Nagelkerke R^2.

| Outcome: |  | Hospitalisation |  |  | ICU^a^ Admission |  |  | Death |  |
| --- | --- | --- | --- | --- | --- | --- | --- | --- | --- |
|  |  | (R^*^ =0.03) |  |  | (R^*^ =0.25) |  |  | (R^*^ =0.405) |  |
| Predictors | aOR | 95% C.I. | *p-value* | aOR | 95% C.I. | *p-value* | aOR | 95% C.I. | *p-value* |
| Time from last vaccine dose (each more day) | 1.003 | (1.002 - 1.004) | <0.001 | 1.002 | (0.998 - 1.008) | 0.328 | 1.005 | (1.003 - 1.008) | <0.001 |
| Gender: Females (vs Males) | 0.465 | (0.378 - 0.574) | <0.001 | 0.219 | (0.081 - 0.590) | 0.003 | 0.483 | (0.313 - 0.743) | <0.001 |
| Age (each more year) | 1.103 | (1.095 - 1.110) | <0.001 | 1.099 | (1.068 - 1.130) | <0.001 | 1.16 | (1.137 - 1.185) | <0.001 |
| Variant: Intermediate (vs Delta) | 0.820 | (0.587 - 1.145) | 0.244 | 0.344 | (0.076 - 1.555) | 0.166 | 0.696 | (0.337 - 1.437) | 0.327 |
| Variant: Omicron (vs Delta) | 0.578 | (0.459 - 0.728) | <0.001 | 0.265 | (0.105 - 0.669) | 0.005 | 0.429 | (0.267 - 0.691) | <0.001 |
